# The switching glare illusion: Appearance and disappearance of glare effect due to figure-ground reversal

**DOI:** 10.1177/20416695231179627

**Published:** 2023-06-08

**Authors:** Risa Yamagata, Kazuho Fukuda

**Affiliations:** Department of Informatics, Graduate School of Engineering, Kogakuin University, Tokyo, Japan; Department of Information Design, Faculty of Informatics, Kogakuin University, Tokyo, Japan

**Keywords:** glare effect, brightness perception, optical illusion, figure-ground reversal, surface completion, depth from luminance gradients

## Abstract

The glare illusion is an illusory perception of brightness enhancement and self-luminosity from a glare pattern, which consists of a central white area and surrounding areas with radial darkening luminance gradients. Here, we report a phenomenon we call “the switching glare illusion.” In this phenomenon, observers experience perceptual alternation in which the glare effect repeatedly appears and disappears or attenuates when the multiple glare patterns are arranged in a grid pattern. This perceptual alternation is caused by a figure-ground reversal in the grid pattern. Since such a phenomenon has not been reported for a single glare pattern, this is caused by arranging multiple glare patterns in a grid. This new finding is worthy for further studies for understanding the mechanisms underlying the glare effect and brightness perception.

This article aims to introduce a phenomenon we call “the switching glare illusion,” where observers’ perception of glare repeatedly appears and disappears to an arrangement of multiple glare patterns. Figure 1a shows the original glare pattern reported by Zavagno (1999). The central white region surrounded by squares with radial luminance gradient induces brightness enhancement and a perception of self-luminosity. This effect of gradient is known as the glare effect, and the induced illusory perception is called the glare illusion. Agostini and Galmonte (2002) showed that the glare pattern enhanced the effect of simultaneous lightness contrast by approximately three times compared to the central white region surrounded by squares with uniform luminance (Figure 1b). Tamura et al. (2016) demonstrated that the brightness enhancement observed in the glare pattern was extremely robust even when the luminance of central region was lower so that it was not perceived as self-luminous.

**Figure 1.**
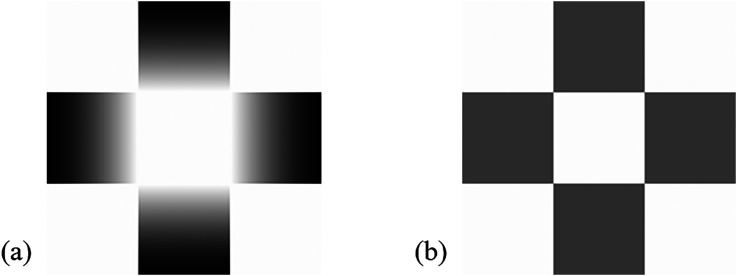
(a) The glare pattern introduced by Zavagno (1999), where a central white region surrounded by squares with radial luminance gradient. (b) The uniform stimulus, where a central white region surrounded by squares with average luminance of the gradient in (a).

Here, we created a new variant of the glare stimulus by arranging multiple glare patterns in a grid, unlike those previous studies with a single glare pattern. This new arrangement shown in Figure 2 induces perceptual alternation involving the appearance and disappearance or attenuation of the glare perception according to multiple perceptual processes: (i) glare effect, (ii) figure-ground assignment (Wagemans et al., 2012), (iii) shape-from-shading (Horn, 1970), and (iv) amodal completion (Nanay, 2018).

**Figure 2.**
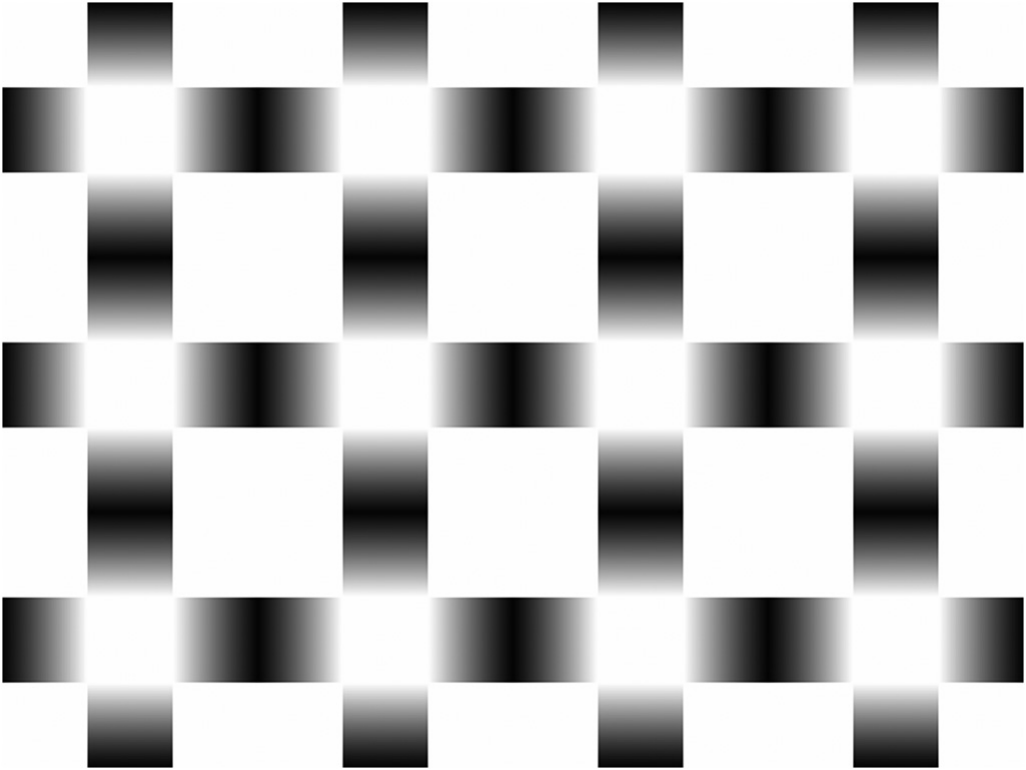
The switching glare stimulus, which induces perceptual alternation with the change in the glare perception. This pattern is created by arranging the glare patterns in a grid.

Figure 3 illustrates the links between the perceptual processes and the possible perceptions. At the first, a grouping process is performed. The glare patterns are grouped as a grid pattern and the others are grouped as a gap. Then, figure-ground assignment process is performed to the groups. When the figure-ground process assigns the grid as a figure and the gaps as a background, then observers perceive strong glare effects from the grid of glare patterns (Figure 3a). On the other hand, when the process assigns the grid as the background behind the gaps and the glare is dissociated from the square by depth, then the strong glare effects disappear. In this case, observers perceive one of the three possible perceptions (Figure 3b to d), which is determined by the combination of the outputs from the following two processes: (i) an amodal completion process which connects the blurred black segments straight or curved behind the square gaps; (ii) a process which assumes the white-to-black luminance gradients as surface color gradients or shading patterns. The combination of straight completions and assuming surface color gradients results in the perception of a texture pattern of blurred black grid lines on the background which are partly occluded by the white square gaps (Figure 3b). The combination of straight completions and assuming shading patterns results in the perception of a horizontal and/or vertical wavy structure in depth on the background by the process of shape-from-shading (Figure 3c). The combination of curved completions and assuming shading patterns results in the perception of white spheres in front of the black colored background by the shape-from-shading process (Figure 3d).

**Figure 3.**
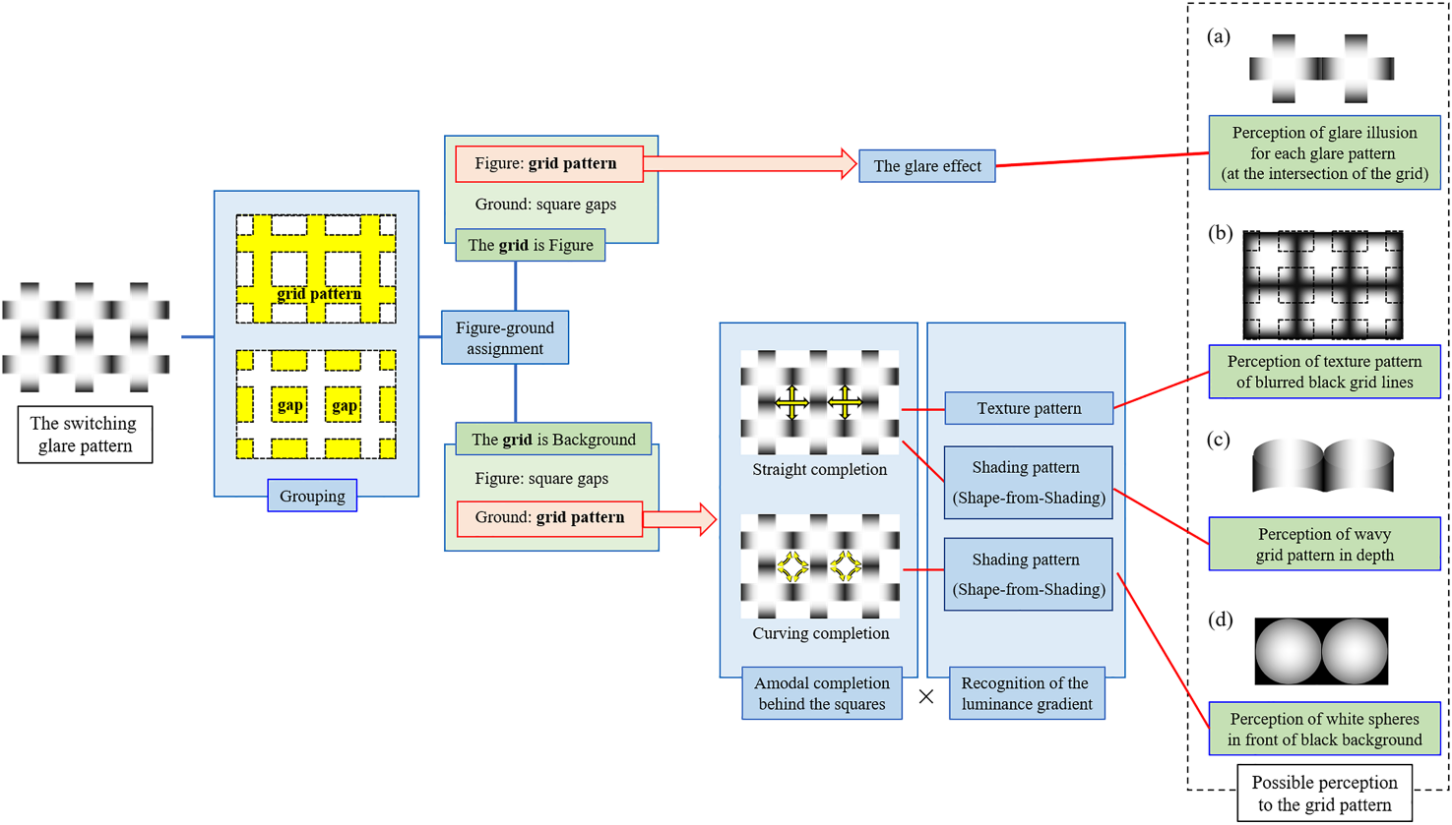
A schematic diagram of possible perceptions and the related processes in the switching glare illusion.

To demonstrate the figure-ground alternation in the switching glare illusion, 13 volunteers (nine males and four females) including the two authors took part in a psychophysical experiment. In the experiment, the switching glare stimulus structured by a grid array of 3  ×  4 glare units shown in Figure 2 was displayed on a 24.1-inch LCD monitor in a dark room. Each unit was 3.1° of visual angle, and the whole stimulus size was 9.1° × 12.2°. Participants observed the stimulus binocularly for 30 s at a distance of 76 cm from the LCD monitor. Participants were instructed to keep pressing the space key while the glares were perceived and to release the key while the glares were disappeared or weakened. Figure 4 shows (a) frequency of perceptual alternation per second and (b) proportion of glare perception to observation duration. Although there was a participant who never perceived the glares, the other participants reported the change of the glare perception with figure-ground reversal.

**Figure 4.**
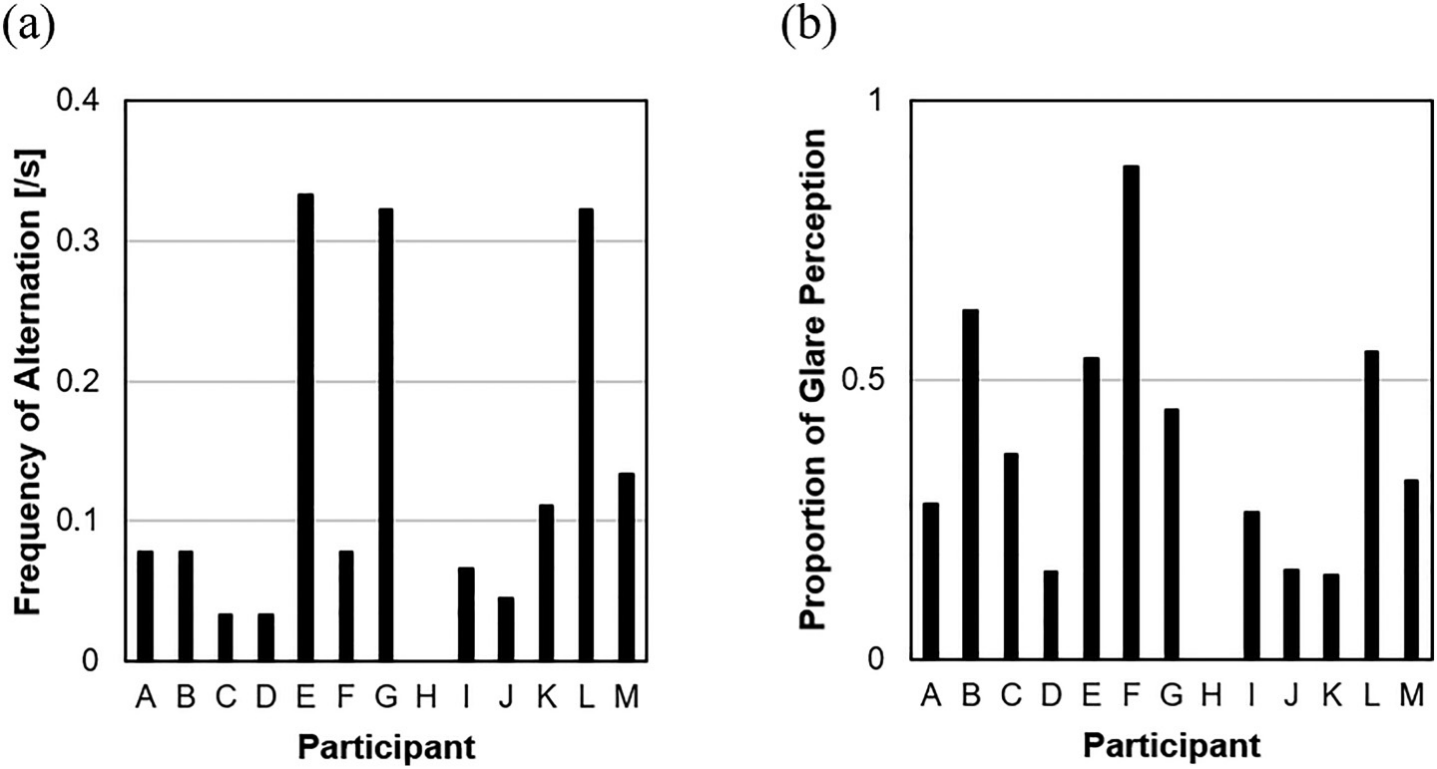
Results of 30 s observations at the switching glare stimulus (Figure 2). Each bar represents the mean of three trials for each participant. (a) Frequency of perceptual alternation per second. (b) Proportion of glare perception to observation duration.

It should be noted that there are some studies considering the glare illusion as a kind of visual phantom, a phenomenon in which illusory light mist appears to lie over a physically homogenous surface (Kitaoka et al., 2006). Kitaoka et al. (2001) explained the perceptual continuation and depth in visual phantoms in terms of perceptual transparency. There is a difference in depth perception between the appearance and disappearance of glare effect in the switching glare illusion. Therefore, this perceptual alternation might suggest that there is a relationship between the glare effect and visual phantom or perceptual transparency.

Similar to the switching glare illusion, the Hermann grid (Hermann, 1870) and its variants also have a grid structure and arise light or dark illusory spots which randomly blink at intersections (Spillmann, 1994). However, unlike those illusions, the switching glare illusion requires the luminance gradients from each intersection to arise illusory glare. Also, it is different from the Hermann grid and other variants that the change in depth perception occurs along with the appearance and disappearance of glare.

Since there are many possible variants of color or luminance in the glare stimuli, the switching glare illusion is also considered to be induced from a variety of figures. Figure 5a shows a low-luminance condition in which the contrast between the central region and the outward of the gradients is low. Brightness enhancements appear and disappear alternately even in this low-luminance condition. Figure 5b shows a variant in which the gradient direction is reversed. Instead of the glare perception, this figure induces visual phantoms surrounding each unit. When the perception switches and the phantoms are disappeared, each black-white-black gradient segment appears as a three-dimensional cylinder. Figure 5c shows a variant in which glare units are arranged diagonally. This figure induces visual phantoms in the oblique directions when the glare perception is present.

**Figure 5.**
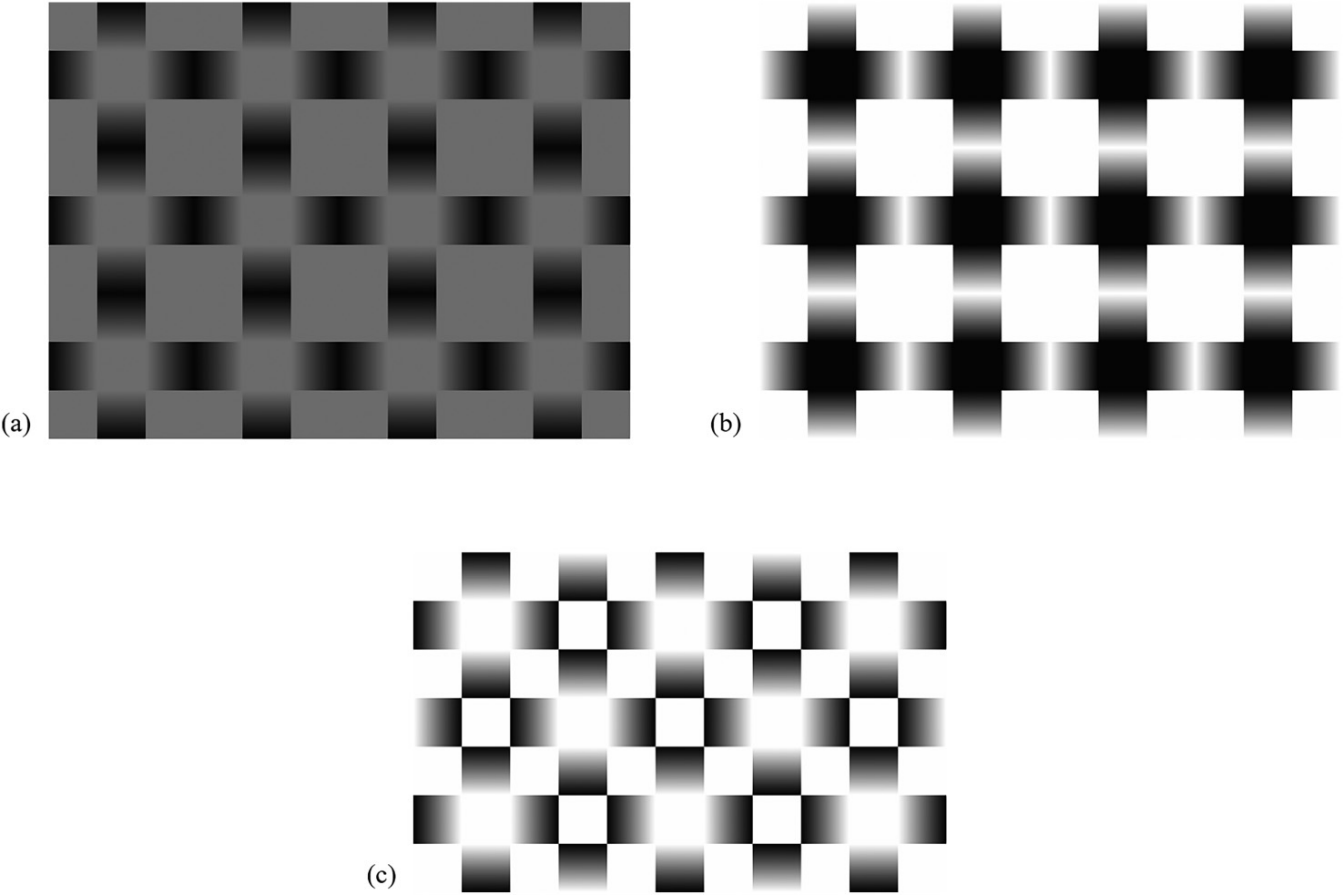
Some variants of the switching glare illusion. (a) The perceptual alternation still occurs in a low-luminance condition. (b) The gradient direction is reversed. Visual phantoms surrounding each unit or three-dimensional cylinders are perceived. (c) In a diagonal arrangement. In the presence of the glare perception, they are obliquely connected and induce visual phantoms.

Although many researchers have considered the glare effects, the perceptual alternation we report in this article seems have not been discussed before. In the original glare effect, it is not the intensity of light but, instead, the two-dimensional pattern that causes a sensation of glare. The switching glare illusion indicates that the appearance and disappearance of the glare effect cannot be explained by only bottom-up processes from the retinal image because the perceptual change in three-dimensional structure caused by a simple figure-ground reversal affects the glare perception. In the switching glare illusion, a dissociation of the grid from the square gaps by depth destroys the glare effect. It suggests that the sensation of glare, the phenomenon that seems to be a low-order response relative to intense light, can be influenced by relatively complex processes of three-dimensional structural perception. Furthermore, some studies pointed out that observing the glare stimuli caused a change in the observer's pupil diameter (Laeng & Endestad, 2012; Suzuki et al., 2019). Consequently, we expect that observing the switching glare illusion may cause the pupil responses along with the changes in the perception of the glare effect and are usable for monitoring the perceptual alternation objectively without verbal reporting.
